# Surgical Outcomes in Patients With Inflammatory Bowel Disease and Primary Sclerosing Cholangitis–Related Liver Disease

**DOI:** 10.14309/crj.0000000000001506

**Published:** 2024-09-27

**Authors:** Sarah Wang, Taaj Raasikh, Florence-Damilola Odufalu

**Affiliations:** 1 Division of Internal Medicine, University of Southern California/Los Angeles General Medical Center, Los Angeles, CA; 2 Division of Gastroenterology & Liver Diseases, Department of Medicine, University of Southern California, Los Angeles, CA

**Keywords:** primary sclerosing cholangitis, inflammatory bowel disease, surgery, colectomy, cholecystectomy

## Abstract

Patients with chronic liver disease have a higher surgical risk compared with those without. For patients with inflammatory bowel disease (IBD), literature has shown that earlier surgical intervention for those with severe IBD has led to better outcomes regarding mortality and remission. For patients who have both IBD and chronic liver disease, management can be complex. The outcomes in this population of patients who undergo surgical intervention have not been thoroughly explored. This case series aims to evaluate surgical outcomes in patients with a diagnosis of both IBD and primary sclerosing cholangitis chronic liver disease.

## INTRODUCTION

It is well known that patients with chronic liver disease have a higher surgical risk compared with those without, and a careful assessment of the risks and benefits of a surgical intervention must be considered.^1,2^ For patients with inflammatory bowel disease (IBD), surgical management may be indicated, particularly if the disease is nonresponsive to medical management or malignancy/neoplasm is identified.^3^ Literature has shown that earlier surgical intervention for those with severe IBD has led to encouraging outcomes with respect to mortality and treatment remission.^4,5^ There is a group of patients who have both IBD and chronic liver disease, and management can often be complex and require a multidisciplinary approach.^2,6^ The postoperative outcomes in this patient population have not been thoroughly explored. This case series aims to evaluate surgical outcomes in patients with IBD and primary sclerosing cholangitis (PSC) chronic liver disease.

## CASE REPORT

**Figure 1.**
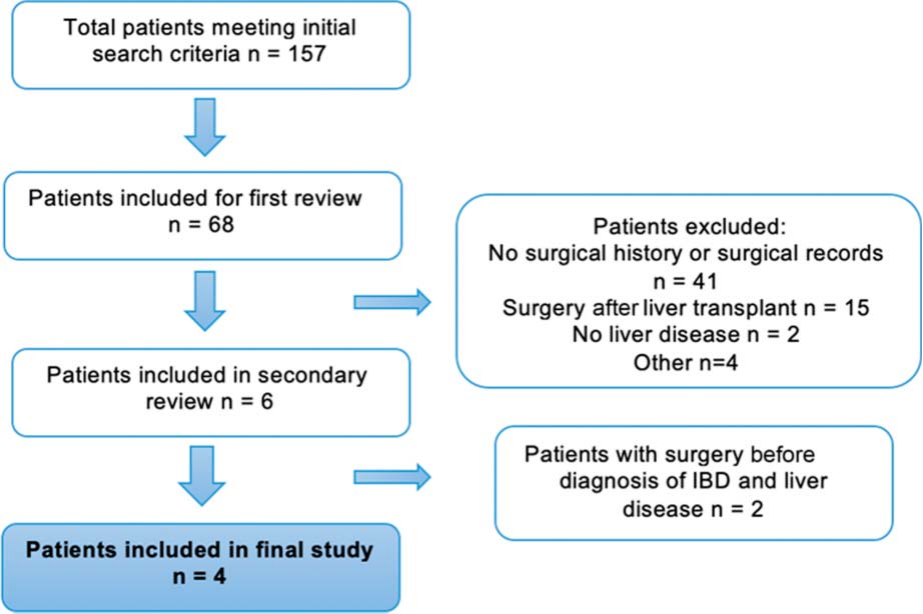
Flowchart for patient selection process.

We performed a case series at our institution, Keck Medical Center of University of Southern California. Our initial search identified 157 patients who were considered for liver transplant for PSC and had an IBD diagnosis. After a detailed review, 4 patients were identified who met criteria and whose data were input and analyzed in a REDCap database (Figure 1).^7,8^

Table 1 details the demographics and outcomes of our patients. All patients were nonsmoking, White males, with an age range of 24 to 61 years. Three (75%) patients were diagnosed with ulcerative colitis (UC), and 1 (25%) was diagnosed with Crohn's disease. Two (50%) patients had received steroids previously, and none were on or had history of biologics for IBD treatment. All patients had PSC as etiology of their liver disease, with Child-Pugh scores (CPS) of either A or B. Two patients underwent cholecystectomies, and 2 underwent colectomies. Three (75%) patients did not have major postoperative complications. We retrospectively reviewed these patients' postoperative courses up until they underwent liver transplantation. One patient had a postoperative liver decompensation resulting in multiorgan failure.

**Table 1.** Patient demographics and disease characteristics

Patient	1	2	3	4
Demographics				
Age	61	24	31	58
Patient alive	Yes	Yes	Yes	No
Ethnicity/race	White	White	White	White
Sex	Male	Male	Male	Male
BMI	23.2	26	20.4	37.7
Tobacco	Never	Never	Former	Never
Alcohol	Never	Former	Current	Never
Marijuana	Never	Former	Current	Never
Other drug use	Never	Never	Never	Never
IBD				
Disease duration (y)	34	5	10	32
Diagnosis	CD	UC	UC	UC
IBD behavior	CD colitis	UC pancolitis	UC pancolitis	UC pancolitis
Clinical remission	Yes	Yes	Yes	Yes
Endoscopic remission	Yes	Unknown	Unknown	Yes
Histological remission	Yes	Unknown	Unknown	Mildly active chronic colitis
Current treatment at time of surgery	Mesalamine	Mercaptopurine, mesalamine	Mesalamine	Mesalamine
Previous treatment	No	Steroids	No	Sulfasalazine, steroids
Liver disease				
Etiology	PSC	PSC	PSC	PSC
Decompensations before surgery	None	None	None	Trace ascites
HCC	No	No	No	No
Indication for liver transplant referral	Biliary strictures	Pruritus	Recurrent cholangitis	Biliary strictures
Surgical history				
Surgery	Cholecystectomy	Cholecystectomy	Segmental colectomy	Transverse colon resection
Indication for surgery	Gallbladder cancer	Cholecystitis	Colon cancer	Tubular adenoma polyp
Year of surgery	2017	2018	2019	2019
Childs-Pugh score	A	A	A	B
Postoperative complications	No	No	No	Ascites/renal failure
Cirrhosis decompensations	No	No	No	Yes

BMI, body mass index; CD, Crohn's disease; HCC, hepatocellular carcinoma; IBD, inflammatory bowel disease; PSC, primary sclerosing cholangitis; UC, ulcerative colitis.

### Patient 1

A 61-year-old man with a 34-year disease duration of Crohn's disease in clinical, endoscopic, and histologic remission with oral mesalamine and a 31-year history of PSC, which over recent years developed into CPS-A cirrhosis complicated by biliary strictures. An endoscopic retrograde cholangiopancreatography to evaluate a gallbladder polyp and biliary stricture diagnosed gallbladder adenocarcinoma. He subsequently underwent a laparoscopic cholecystectomy and 2 weeks later a radical cholecystectomy for residual margins. He did not develop any postoperative complications or liver decompensations.

### Patient 2

A 24-year-old man with a 5-year disease duration of ulcerative pancolitis, in clinical remission with 6-mercaptopurine and oral mesalamine and a 5-year history of CPS-A PSC cirrhosis complicated by biliary strictures and pruritus. He underwent laparoscopic cholecystectomy for diagnosis of cholecystitis. There were no postoperative complications or liver decompensations.

### Patient 3

A 31-year-old man with a 10-year disease duration of ulcerative pancolitis in clinical remission with oral mesalamine and a 5-year history of CPS-A PSC cirrhosis. The patient had fragmented medical care and went over 5 years without undergoing surveillance colonoscopy. A colonoscopy showed a right-sided mass with biopsies positive for colon adenocarcinoma, with otherwise Mayo 0-1 mucosa in remainder of the colon. He underwent a right hemicolectomy for definitive management. He recovered from surgery without liver decompensation.

### Patient 4

A 58-year-old man with a 32-year disease duration of ulcerative pancolitis presented to Keck Medical Center. Previously, he had mild to moderately active disease on sulfasalazine, so therapy was changed to optimized mesalamine dosing. With oral and rectal mesalamine he had been in clinical and endoscopic remission, with mildly active chronic colitis in the left colon on histology. He had a 5-year history of CPS-B PSC cirrhosis, complicated by high-grade biliary strictures. Given biliary strictures requiring frequent endoscopic retrograde cholangiopancreatographies, he was listed for liver transplantation. His routine surveillance colonoscopy revealed a 3- to 4-cm transverse colon polyp that was unsuccessfully resected endoscopically. He subsequently underwent a combined endolaparoscopic wedge resection with postoperative course complicated by anasarca and hepatic infarct. He developed acute severe abdominal pain and septic shock of unclear origin and subsequently passed from multisystem organ failure.

## DISCUSSION

In this case series, we observed patients with IBD and PSC CPS-A cirrhosis tolerate abdominal surgical intervention without complication. Laparoscopic cholecystectomy has been studied in patients with CPS-A and CPS-B cirrhosis and has been shown to be an effective and safe treatment for symptomatic disease.^10^ However, a previously published study showed that patients with IBD who undergo cholecystectomy are at increased risk of postop complications.^9,11^ Patients 1 and 2 underwent cholecystectomy for cholecystitis and gallbladder cancer both recovered well, suggesting that patients who undergo cholecystectomy with IBD and CPS-A PSC cirrhosis can have positive outcomes. Patients 1 and 2 also had well-controlled biologic-naive IBD, which may have also played a role in the favorable surgical outcomes.

When assessing colonic surgery, patients 3 and 4 had vastly different outcomes. Patient 3 with CPS-A cirrhosis and UC in clinical remission tolerated a right hemicolectomy without decompensation. Patient 4 with UC and CPS-B cirrhosis who underwent a laparoscopic wedge resection had acute liver decompensation and eventually died. Although his UC was in clinical and endoscopic remission, he had CPS-B cirrhosis, which has a 30% mortality rate after abdominal surgery.^12,13^

Given that nearly 5% of patients with IBD will also develop or have chronic liver disease, it is important to explore the surgical outcomes for this population.^14^ Our case series is novel in investigating the outcomes of patients with IBD and PSC liver disease who underwent abdominal surgery. For our patients with well-controlled IBD and CPS-A liver disease, they were able to tolerate surgical intervention without postoperative complications.

This study begins to touch on the complexity of managing high-risk patients with cirrhosis and concurrent IBD who need surgical intervention. Future studies examining the outcomes of cirrhotic patients with IBD may provide further insight and guidance on preoperative and postoperative management.

This case series demonstrated uncomplicated surgical outcomes in patients with both CPS-A PSC cirrhosis and well-controlled IBD undergoing planned abdominal surgery. The observed adverse outcome in one patient with CPS-B cirrhosis and IBD is telling and stresses the challenges and vulnerability in this patient population. Caution and careful evaluation should be given to these patients with planned intra-abdominal surgery.

## DISCLOSURES

Author contributions: conception and design: all authors. Administrative support: D. Odufalu. Provision of study materials or patients: D. Odufalu and T. Raasikh. Collection and assembly of data: T. Raasikh and S. Wang. Data analysis and interpretation: T. Raasikh and S. Wang. Manuscript writing: All authors. Final approval of manuscript: all authors. D. Odufalu is the article guarantor.

Acknowledgments: The authors thank Dr. Jeff Kahn (Keck Medical Center of USC) for providing information for the patient database.

Funding support: This study was supported by a grant from the Color of Crohn's and Chronic Illness (COCCI). F. Odufalu is on the editorial board for Inflammatory Bowel Diseases Journal and has served as a consultant, advisory board member, and/or speaker for AbbVie, Bristol Myers Squibb, EpiQ, Janssen Inc, Pfizer, and Takeda. S. Wang and T. Raasikh do not have any financial disclosures.

Informed consent was obtained for this case report.

Ethical statement: The authors are accountable for all aspects of the work in ensuring that questions related to the accuracy or integrity of any part of the work are appropriately investigated and resolved. The study was conducted in accordance with the Declaration of Helsinki (as revised in 2013). The study was approved by the institutional review board of Keck Medical Center at the University of Southern California (IRB ID: HS-23-00622), and individual consent for this retrospective analysis was waived.
